# *Trichoderma*-amended biofertilizer stimulates soil resident *Aspergillus* population for joint plant growth promotion

**DOI:** 10.1038/s41522-022-00321-z

**Published:** 2022-07-12

**Authors:** Xinnan Hang, Lingxue Meng, Yannan Ou, Cheng Shao, Wu Xiong, Nan Zhang, Hongjun Liu, Rong Li, Qirong Shen, George A. Kowalchuk

**Affiliations:** 1 Jiangsu Provincial Key Lab of Solid Organic Waste Utilization, Jiangsu Collaborative Innovation Center of Solid Organic Wastes, Educational Ministry Engineering Center of Resource-saving fertilizers, Nanjing Agricultural University, Nanjing, 210095 Jiangsu China; 2 Ecology and Biodiversity Group, Department of Biology, Institute of Environmental Biology, Utrecht University, 3584 CH Utrecht, the Netherlands

**Keywords:** Microbial ecology, Next-generation sequencing

## Abstract

Application of plant growth-promoting microbes (PGPMs) can contribute to sustainable agricultural ecosystems. From a three-year field experiment, we already found that the addition of *Trichoderma* bio-organic fertilizer (BF) significantly improved crop growth and yield compared to the application of organic fertilizer (OF). Here, we tracked the responses of soil bacterial and fungal communities to these treatments to find the key soil microbial taxa that contribute to the crop yield enhancement. We also examined if bacterial and fungal suspensions from resulting soils could improve plant growth upon inoculation into sterilized soil. Lastly, we isolated a number of fungal strains related to populations affected by treatments to examine their role in plant growth promotion. Results showed that consecutive application of BF impacted soil fungal communities, and the biological nature of plant growth promotion was confirmed via pot experiments using γ-sterilized versus none-sterilized soils collected from the field. Soil slurry experiments suggested that fungal, but not bacterial communities, played an important role in plant growth promotion, consistent with the results of our field experimental data. Fungal community analysis of both field and slurry experimental soils revealed increases in specific resident *Aspergillus spp*. Interestingly, *Aspergillus tamarii* showed no plant growth promotion by itself, but strongly increased the growth promotion activity of the *Trichoderma* amendment strain upon their co-inoculation. The effectiveness of the fungal amendment appears to stem not only from its own action, but also from synergetic interactions with resident fungal populations activated upon biofertilizer application.

## Introduction

Agricultural intensification has resulted in an increased production of staple crops including cereal and commercial crops, leading to greater food security for an increasing world population^1^. However, it also comes with large environmental costs, due to the use of high amounts of detrimental agrochemicals^2,3^. Thus, there is an urgent need to develop more environmentally friendly means for reliable high crop yields. Plant growth-promoting microbes offer new and promising strategies to confront sustainability issues in agriculture^4–6^, but the mechanisms that determine the success of such measures are often lacking.

Numerous researchers have studied the functions of plant growth-promoting rhizobacteria (PGPRs), such as synthesis of phytohormones to increase elemental nutrient uptake^7^, direct and indirect effects on insect pest resistance^8^, synthesis of antimicrobial metabolites^9^ and triggering induced systemic resistance^10^. Similarly, the plant growth promotion functions of plant growth-promoting fungi (PGPFs) include the production of phytohormones, mineralization of organic matter, solubilization of unavailable soil-bound nutrients, activation of induced systemic resistance, and protection from biotic and abiotic stresses^11,12^. Thus, the utilization of plant growth-promoting microbes (PGPMs) for enhancing crop production represents a viable alternative to traditional intensive agricultural practices, and the delivery of such plant probiotics via for instance bioorganic fertilizers (BF) has proven particularly effective in improving soil microbial functionality^13^. Although potentially effective, the mechanisms driving the success of such BF applications are generally not well described, due to the multiple modes of action that are possible, including direct activities attributed to PGPFs and indirect effects derived from impacts on the resident microbiome^8,14–16^. Within the soil microbiome, previous studies have shown that specific microbial groups related to plant growth promotion may be stimulated by the application of BF containing PGPRs (such as *Bacillus sp*., *Pseudomonas sp*., etc.)^17,18^. Such results suggest that strategies that stimulate the activities of the soil-borne microbial groups may help improve the effectiveness and generality of PGPFs amendments via BF application. However, few studies have been able to disentangle the direct versus indirect impacts of PGPF inoculation.

We conducted a three-year greenhouse experiment with four treatments: Ctrl, soil amended with no fertilizer; CF, soil amended with chemical fertilizer; organic fertilizer (OF), soil amended with OF and BF, soil amended with bio-organic fertilizer, and found that different fertilizer patterns induced different cucumber yields^19^. We also observed microbe-consuming protists can indirectly increase crop yield by regulating the microflora of fungi and bacteria in the soil^19^. However, as the direct influencer of fertilization and direct driver of crop yield, the community characteristics and microbiological mechanism of fungi and bacteria are still unclear. From the field experiment, we further observed that the BF (*Trichoderma* bio-organic fertilizer) significantly increased cucumber yield compared to OF, providing the opportunity to explore the mechanisms driving the success of such BF applications.

In this study, these two treatments were selected and we tried to disentangle the direct versus indirect underlying mechanisms of PGPF inoculation involved in promoting cucumber growth over a period of six seasons. The main objectives of this study were (1) to evaluate whether the enhanced yield after BF application was principally due to physicochemical changes or biological factors; (2) to reveal the extent to which improved plant performance was due to PGPF-induced changes in the resident bacterial versus fungal community; and (3) to decipher which components of the soil microbiome were potentially responsible or microbes are the key taxa induced by particular soil practices and confirm their real functions though verification test. We hypothesized that *Trichoderma* bio-organic fertilizer mainly affected crop growth and yield by regulating soil fungi, and this would be verified by high-throughput sequencing data analysis and soil suspension pot experiment. We further hypothesized that the increase in yield was caused by the combined action of *Trichoderma* and the microorganisms recruited by it, rather than the individual action.

## Results

### Cucumber yield and soil physicochemical versus biological factors on cucumber growth

**Fig. 1:**
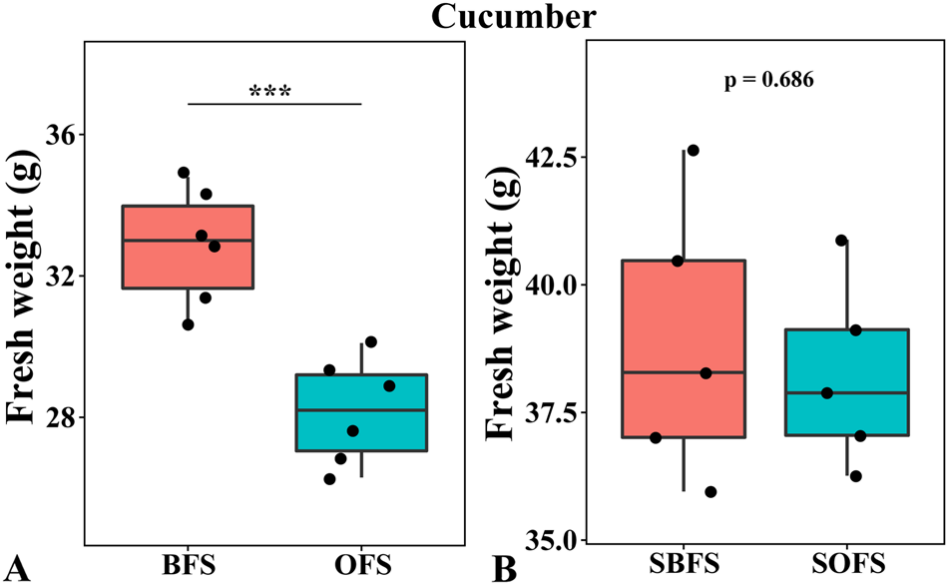
Effects of different treatments on cucumber growth. Growth of cucumber cultivated with none-sterilized soil (**A**) and γ-sterilized soil (**B**) of different treatments. Soil collected from OF and BF treatments defined as OFS and BFS. Soil collected and then γ-sterilized from OF and BF treatments defined as SOFS and SBFS. An asterisk indicates a statistically significant difference (Turkey’s test, *p < 0.05, **p < 0.01, ***p < 0.001) between OFS and BFS treatments.

As described in our previous study^19^, there was no significant difference (t-test, *P* > 0.05) in cucumber yield observed between the OF and bio-organic fertilizer (BF) treatments in the first season. However, cucumber yields in the BF treatments were significantly (t-test, *P* < 0.05) higher than OF since the second season. Compared to the OF treatment, the BF treatment increased yield by 8.7, 16.7, 14.8, 19.1, 13.1, and 20.7% from one to six seasons, respectively. In order to verify whether physicochemical factors or biological factors promoted cucumber growth in the field, we conducted a pot experiment with soil sterilization and found that cultivating plants with the soil collected from field, the growth of plants in BFS was significantly higher than that of OFS both on crop cucumber (Fig. 1A) and *Arabidopsis* (Supplementary Fig. 1A). However, after γ-ray sterilization, there was no significant difference in the plant growth of cucumber (Fig. 1B) and *Arabidopsis* (Supplementary Fig. 1B) between these two treatments. These results indicated the biological nature rather than physicochemical nature of the bio-organic fertilizer impacts on plant growth across these two plant species.

### Microbial diversity and community structure in field and pot experiments

The diversity (Shannon), richness (Chao1), and evenness (Pielou) of bacteria (Supplementary Fig. 2A) and fungi (Supplementary Fig. 2B) were not influenced by treatments in bulk soil. The principal coordinate analysis (PCoA) based on the Bray–Curtis distance of field experiment (Supplementary Fig. 3) showed that seasons had a significant impact on the structure of both bacteria (*R*^2^ = 0.435, *P* < 0.001) and fungi (*R*^2^ = 0.583, *P* < 0.001) while the treatments had a significant effect on the community structure of fungi (*R*^2^ = 0.072, *P* < 0.05) but not on the bacteria (*R*^2^ = 0.026, *P* = 0.468). Moreover, the PCoA analysis of pot experiment also revealed that the community structure of fungi (*R*^2^ = 0.44, *P* < 0.001) was more affected by treatments than bacteria (*R*^2^ = 0.12, *P* = 0.22) (Supplementary Fig. 4). The results indicated that the addition of *Trichoderma* only significantly affected the structure of fungal community in both field and pot experiments.

### Effects of fungal communities versus bacterial communities on cucumber yield

**Fig. 2:**
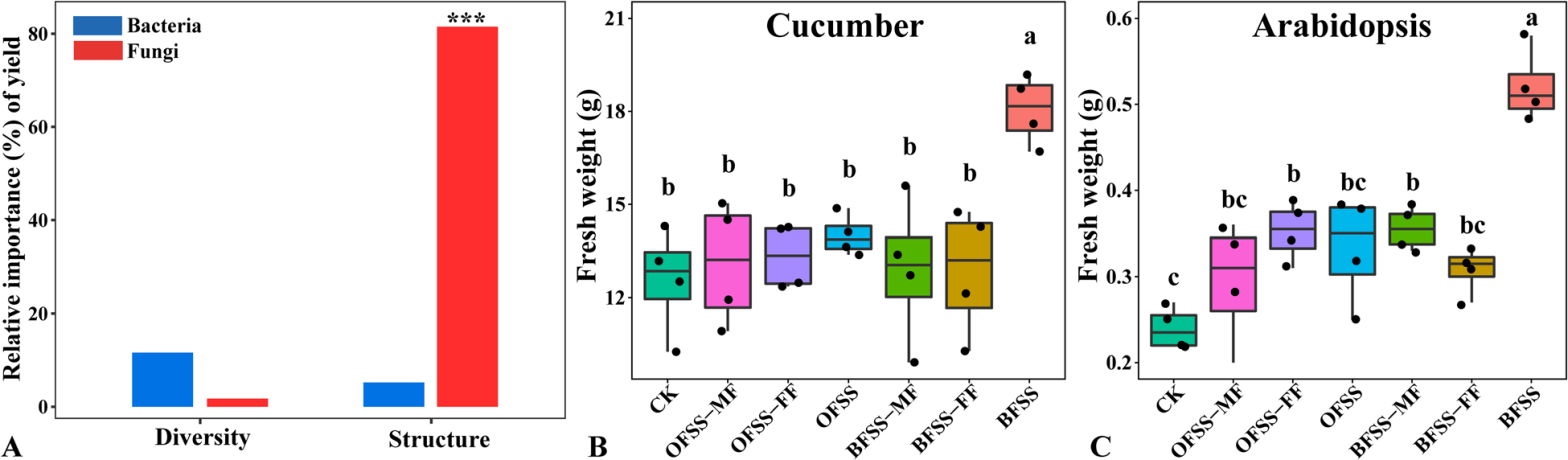
Effects of soil bacterial and fungal community on cucumber growth. Relative importance of diversity and structure of fungal and bacterial community on cucumber yield (**A**) and effects of different soil slurries on cucumber (**B**) and *Arabidopsis* growth (**C**). In A, ***P < 0.001. Statistical significance for relative importance was calculated by multiple regression using linear models. BFSS and OFSS represent bacterial and fungal suspensions from the *Trichoderma*-amended bio-organic fertilizer and organic fertilizer treatments, respectively. BFSS-FF and OFSS-FF indicate bacterial suspensions from these same treatments, while BFSS-MF and OFSS-MF represent microbe-free suspensions from these same treatments. BBFSS and BOFSS represent bulk soils samples from BFSS and OFSS, respectively, while RBFSS and ROFSS indicate rhizosphere samples from these same treatments. Bars with different letters indicate significant differences as defined by Turkey’s test (*P* < 0.05).

In field experiment, the generalize linear model analysis indicated that the structure of fungal community-contributed most to the cucumber yield (81.5%) rather than fungal diversity (15.8%) and bacterial community diversity (2.37%) and structure (9.98%) (Fig. 2A). In pot experiment, when transferring bacterial and fungal microbiomes using both cucumber (Fig. 2A) and *Arabidopsis* (Fig. 2B) as test plants, there was no significant difference between the OFSS-MF (without bacteria and fungi), OFSS-FF (with bacteria but without fungi), OFSS (with bacteria and fungi), BFSS-MF (without bacteria and fungi) and BFSS-FF (with bacteria but without fungi) treatments, except in the case of transfer of the fungal fraction from the BF treatment (BFSS). This result suggests that the fungal component of the BF-treated soil contributed most to plant growth promotion.

### Key fungi affecting cucumber yield

**Fig. 3:**
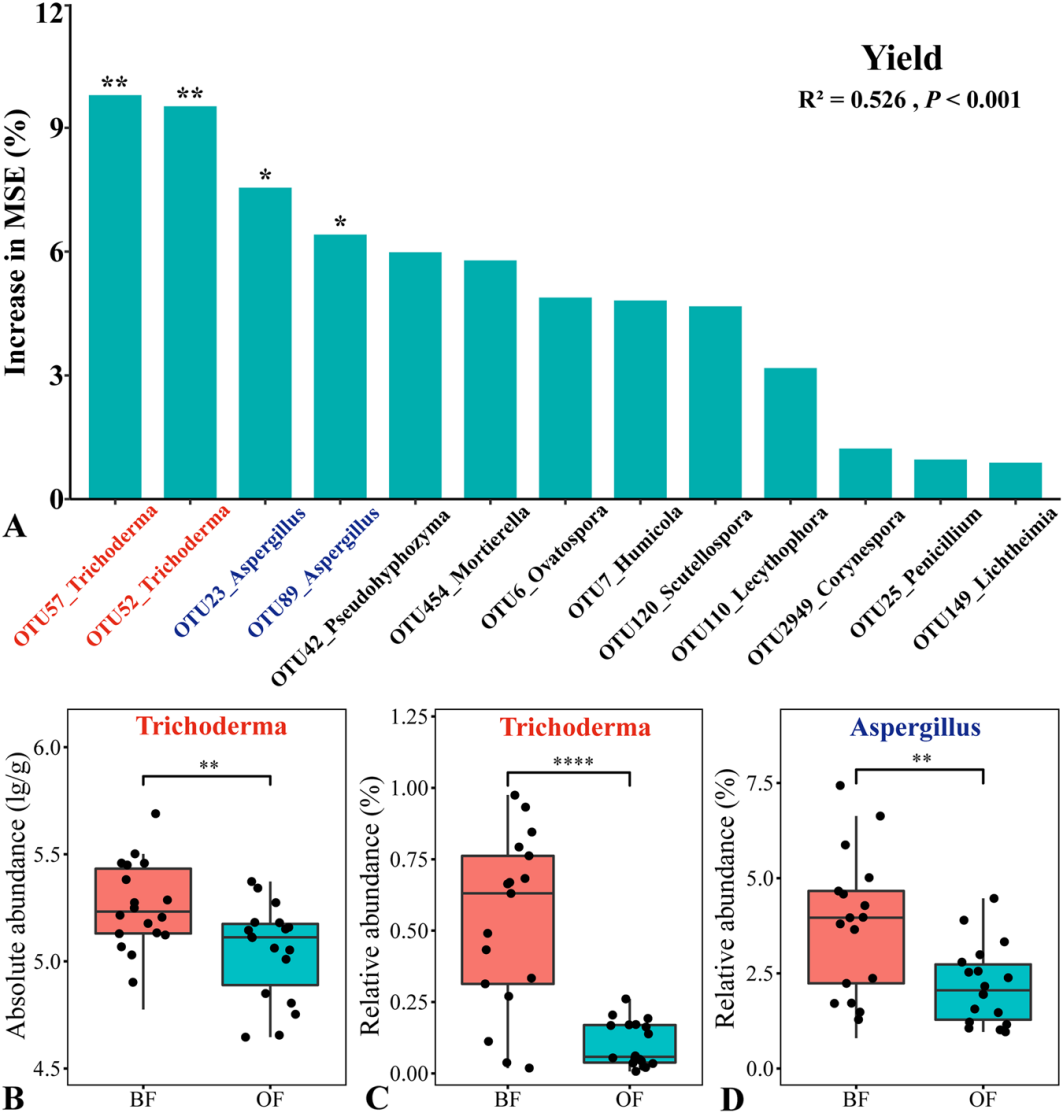
Effects of responses fungal OTUs on cucumber growth. Random forest (RF) means predictor importance (percentage of increase of mean square error) of different fungal OTUs on cucumber yield (**A**). *Trichoderma* absolute abundance (**B**) and relative abundance (**C**) and *Aspergillus* relative abundance (**D**). The accuracy importance measure was computed for each tree and averaged over the forest (5000 trees). Percentage increases in the MSE (mean squared error) of variables were used to estimate the importance of these predictors, and higher MSE% values imply more important predictors. Significance levels are as follows: *P < 0.05 and **P < 0.01. MSE, mean squared error. Absolute abundance was determined by Real-Time PCR quantification with *Trichoderma* specific primer and relative abundance was determined by high-throughput sequencing.

From field experiment, random forest model (RF) was performed to analyze and compare the relative importance of different fungal species (OTUs) in different treatments on cucumber yield (Fig. 3A) and the results revealed that *Trichoderma* (OTU52 and OTU 57) and *Aspergillus* (OTU23 and OTU89) were the key fungi that contributed to the difference of cucumber yield. The result of real-time PCR quantification showed that the *Trichoderma* abundance of BF was higher than that of OF (Fig. 3B). Meanwhile, we found that BF had higher relative abundance of both *Trichoderma* (Fig 3C) and *Aspergillus* (Fig. 3D) than OF from the results of high-throughput sequencing. In pot experiment, stamp analyses based on Benjammini–Hochberg FDR of top most abundant bulk and rhizosphere fungal genera are shown in Supplementary Fig. 5 and the BFSS treatment showed significantly higher relative abundance of *Aspergillus* in both bulk and rhizosphere soil.

### Fungal isolates and plant growth-promoting ability

Seventy fungal strains from each bulk soil of OF and BF were randomly screened (Supplementary Table 1). Three of the strains from the BF treatment were identified as *Trichoderma harzianum*, while no isolates of this species were recovered from the OF treatment. The majority of isolates from both OF (40) and BF (53) treatments were identified as *Aspergillus* strains, with *A. niger* being the most dominant species (31/40 and 29/53 of *Aspergillus* isolates from the OF and BF soils, respectively). Interestingly, a total of 17 *A. tamarii* strains were recovered from the BF treatment, yet only 4 isolates of this species were recovered from the OF soil. Besides, five *Fusarium* strains were isolated from the OF treatment, while only one was isolated from the BF treatment. Moreover, in the third-season field experiment, the relative abundance of *Fusarium* in OF treatment was significantly higher than that in BF treatment (Supplementary Fig. 6). These results indicate that *Trichoderma* bio-organic fertilizer can reduce the abundance of *Fusarium* in soil.

**Fig. 4:**
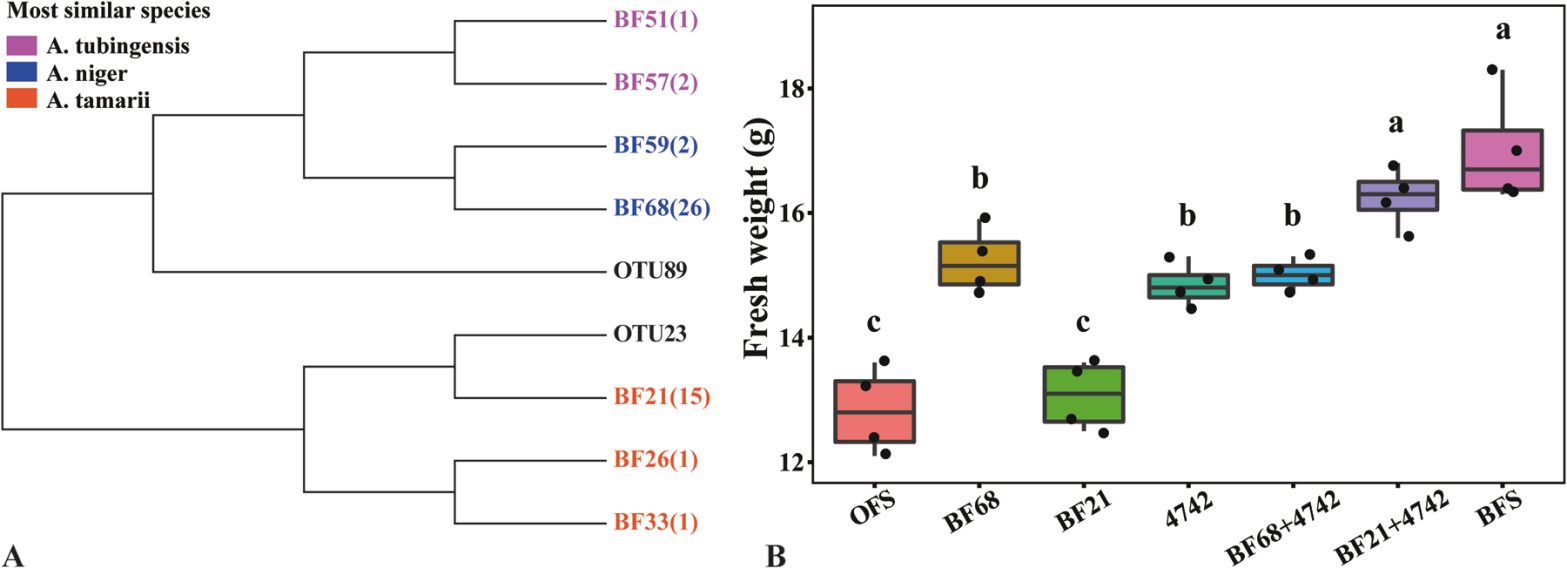
Effects of combined responses Aspergillus species and Trichoderma guizhouense NJAU 4742 on cucumber growth. Hierarchical clustering of the ITS/5.8S rRNA gene sequences of the isolated *Aspergillus* strains from BF treatment and the core *Aspergillus* OTUs sequences (**A**) and effects of selected strains on cucumber growth when inoculated alone or in combination with *Trichoderma guizhouense* NJAU 4742 (**B**). In B, OFS and BFS indicate soil collected from the OF and BF treatments, respectively. Bars with different letters indicate significant differences as defined by Duncan’s test (*P* < 0.05).

Hierarchical clustering of the ITS/5.8S sequences for isolates from BF treatment and responsive *Aspergillus* OTUs revealed that BF21 was the most phylogenetically similar to OTU23 and BF68 was the most phylogenetically similar to OTU89 (Fig. 4A). Moreover, the results of sequence alignment revealed that BF21 displayed 99% sequence identity with the responsive OTU_23 and BF68 displayed 99% sequence identity with the responsive OTU_89. Based on these results, we selected BF21 (*A. tamarii*) and BF68 (*A. niger*), along with *Trichoderma guizhouense* NJAU 4742 (4742) for further testing in a pot experiment to determine plant growth-promoting capabilities when inoculated to soil in isolation or as co-cultures (Fig. 4B). Compared to OFS, the BF68 and 4742 treatments significantly promoted cucumber growth, while the BF21 treatment showed no significant plant growth promotion. There were also no significant differences across the BF68, 4742, and BF68 + 4742 treatments. Interestingly, the BF21 + 4742 treatment significantly increased cucumber growth compared to the BF21 and 4742 treatments. Furthermore, the growth promotion of the BF21 + 4742 treatment was comparable to the BFS treatment, indicating that simple combination of strains 4742 and BF21 was as effective as the whole BF soil microbiome.

## Discussion

In accordance with previous studies^20,21^, *Trichoderma guizhouense* NJAU 4742 (NJAU 4742)-amended bio-organic fertilizer (BF) significantly increased cucumber yield under field conditions as compared to OF application, and this fungal species could be detected in the BF-treated soils. Moreover, soil collected from the BF treatment not only enhanced cucumber growth, but also significantly promoted *Arabidopsis* growth, compared to soil collected from OF treatment, and the biological nature of the growth promotion was confirmed by controls using γ-sterilized soils. The analysis also indicated that the physicochemical properties were mainly affected by the types of fertilization, which was similar to previous research^22^, and the input of *Trichoderma* has little promotion on the physicochemical properties. Thus, similar to previous finding^23,24^, the changes induced by BF application proved to be more important in affecting microbial community to drive plant performance, and repeated BF inputs resulted in positive impacts over time.

Microbial community analysis showed that there was no significant difference in the diversity and richness of the communities of both bacteria and fungi among different treatments. Furthermore, we found that BF mainly influenced fungal rather than bacterial community structure in the field, a result that is in line with previous findings in response to *Trichoderma harzianum* T-E5 application^21^. Meanwhile, generalized linear model analysis indicated that fungal community structure was the main driver of cucumber yield changes. In total, the results from our field experiment suggested that BF-induced changes in fungal communities were more important than changes in the bacterial community for promoting cucumber growth.

Using our soil slurry experiment, we could directly demonstrate that the fungal community resulting from the BF treatment could confer not only cucumber plant growth promotion, but also the growth promotion of *Arabidopsis*. Such conferral of growth promotion was not achieved by transfer of the bacterial community. Using the same approach, Møller et al.^25^ also demonstrated a more dominant role of fungi as compared to bacteria in the degradation of recalcitrant litter. We evaluated the importance of various fungal genera to cucumber yield by random forest and the result revealed that *Trichoderma* and *Aspergillus* were the key fungi related with yield. Our previous study in the same filed experiment revealed that the relative abundances of these two genera were positively correlated with microbe-consuming protistan OTU (cercozoan OTU124) which positively correlated with crop yield^19^. Here, compared with other treatments, BF had higher absolute and relative abundance of *Trichoderma*, interestingly, our field and soil slurry experiments both indicated that the relative abundance of *Aspergillus* increased in response to the BF treatment, suggesting that NJAU 4742 can enrich the soil microbiome for indigenous members of this fungal genus.

To gain more direct evidence for the impacts of fungal populations in contributing to plant growth promotion, we obtained fungal isolates from the OF and BF soils and tested specific strains for their growth-promoting activities. In addition to indigenous fungi, we also recovered three *Trichoderma harzianum* isolates in BF treatment (0 from the OF treatment), supporting our high throughput sequence data and previous studies^20^ that the biological agent of the BF treatment was able to establish itself in the soil. Moreover, five *Fusarium* strains were isolated from the OF treatment, as opposed to just one from the BF treatment, suggesting that the BF treatment could suppress the relative abundance of *Fusarium*, a result that is also in line with our fungal community sequencing results. The majority of our isolates belonged to the genus *Aspergillus* spp., which is not surprising given the high abundance and wide distribution of this genus in soil environments^26^. A number of our fungal isolates showed high levels of similarity to OTUs detected via high-throughput sequencing. Upon soil inoculation, BF68 was able to improve cucumber growth to a similar extent as observed for NJAU 4742 inoculation, and co-inoculation of these strains did not provide any further improvement in plant growth (Fig. 4). However, neither of these treatments was as effective as the BFS (nature soil from BF treatment in the field) treatment in promoting plant growth. Interestingly, soil inoculation with BF21 did not contribute plant growth enhancement. Interestingly, when this strain was combined with NJAU 4742 and BF21, strong plant growth promotion was observed, yielding a growth promotion level that was similar to the full BFS treatment. A previous study has shown that co-cultivation of *Trichoderma reesei* RutC30 with three black *Aspergillus* strains could lead to synergistic effects on hydrolysis of pretreated wheat straw^27,28^. Similar synergistic effects of soil bacterial activities have also previously been described. For instance, Berendsen et al.^28^ observed an increase in three bacterial populations under conditions of downy mildew disease. Although none of these enriched species showed an impact on disease suppression alone, inoculation with all three did (provide effective disease suppression). In our study, the level of plant growth promotion in the BF treatment appears to be the consequence of the direct action of NJAU 4742, which enhanced activity of indigenous fungal populations with potential plant growth-promoting activities, and the enrichment of fungal populations that are only effective in plant promotion in combination with the inoculated biocontrol strain. Recent studies have demonstrated a range of mechanisms for plant growth promoting mediated by *Trichoderma spp*., such as mycoparasitism^29^, antibiotic activity^30^, induction of systemic resistance to pathogens in plants^11^, and enhanced nutrient supply to the plant^31^. Additionally, similar results have shown that the disease suppression effectiveness of *Trichoderma* enriched bio-fertilizer was attributed to a combination of the actual antagonistic activities of the inoculated bio-control *Trichoderma* strain as well as the promotion of beneficial microbial groups already resident in the soil^14^. Here, we expand the range of plant growth promotion mechanisms to include the recruitment of indigenous fungal strains that cooperate in achieving plant growth promotion.

In this study, the application of *Trichoderma-*amended bio-organic fertilizer effectively and progressively enhanced cucumber yield. Bio-organic fertilizer application resulted in shifts in the soil microbiome, especially within the fungal community, and transplantation of fungal communities via soil slurries conferred the same growth promotion capabilities as BF soil from the field. Specific fungal populations were induced in BF treatment, with *A. tamarii* displaying highly synergistic interactions with NJAU 4742 in achieving reinforced plant growth promotion. Thereby, plant growth-promoting activity is achieved by a combination of direct and indirect mechanisms, including interaction with stimulated indigenous fungal populations that fail to improve plant growth on their own. Thus, the effectiveness of soil microbiota manipulation seems to be a combination of the actual plant growth promotion activities of the inoculated beneficial agents as well as the promotion of beneficial microbial groups already resident in the soil. We propose that these additional beneficial effects should also be kept in mind in the design of bio-organic fertilizers and their potential utility in sustainable agriculture for plant growth promotion.

## Methods

### Field description, experimental design, and soil sampling

The field description, experimental design, and soil sampling had been described in detail in our previous study^19^. In the three-year field experiment (planted twice per year), the BF (*Trichoderma* bio-organic fertilizer) significantly increased cucumber yield compared to OF. Each fertilizer treatment was established in a randomized complete block design with three replicates for each treatment. In this study, these two treatments were selected to disentangle the direct versus indirect underlying mechanisms of PGPF inoculation involved in promoting cucumber growth over a period of six seasons.

### Pot experiments to distinguish biological versus physicochemical factors contributing to yield enhancement

Soils were collected from BF and OF treatments in the third-season field experiment. To distinguish biological versus physicochemical impacts of the treatments on yield enhancement, we included sterilized soil controls in our experimental design, with the resulting 4 treatments: BFS, soil collected from BF; OFS, soil collected from OF; SBFS, soil collected from BF and γ-sterilized according to Xun et al.^32^; SOFS, soil collected from OF and γ-sterilized. Each treatment got six replicates. Cucumber and *Arabidopsis* were selected to test for impacts on plant performance. Cucumber and *Arabidopsis* were planted in pots containing 1000 g (dry weight, DW) and 40 g (DW) of soil, respectively. A. thaliana ecotype Columbia (Col-0) plants were grown in a growth chamber with a 16 h light period (250 μmol/m^2^/s illumination)/8 h dark period at 23/18 °C in this study, and cucumber plants were grown in the greenhouse. Plant fresh weight was determined after three weeks for cucumber and four weeks for *Arabidopsis*.

### Pot experiments to determine bacterial and fungal contributions to plant yield enhancement

In order to determine the third-season field soil bacterial and fungal contributions to plant yield enhancement, a second pot experiment was established in which filtered or unfiltered soil slurries were inoculated to sterilized soil. The experiment contained 7 treatments (Table 1): CK, soil amended with sterile Hoagland nutrient solution; BFSS, soil amended with soil slurry prepared from BF according to Badri et al.^33^; BFSS-FF, soil amended with fungi-free soil slurry from BFSS by filtering through 3 μm pore size membrane as described by Møller et al.^25^; BFSS-MF, soil amended with microbe-free soil slurry from BFSS by filtering through 0.45 μm pore size membrane as described by Badri et al.^33^; OFSS, soil amended with soil slurry prepared from OF treatment; OFSS-FF, soil amended with fungi-free soil slurry prepared from OFSS; and OFSS-MF, soil amended with microbe-free soil slurry prepared from OFSS. Each treatment got four replicates. Cucumber and Arabidopsis were planted in 40 and 10 g γ-sterilized quartz sand and vermiculite (m/m = 2:1), respectively, that were amended with the soil slurry treatments described above. Fresh weight was determined as in first pot experiment, and 4 bulk and 4 rhizosphere soil samples of cucumber were collected from the OFSS and BFSS treatments as described above.

**Table 1:** Preparation of different soil slurry for pot experiment.

Treatment	Soil origin	Microbe	Operation
CK	None	None	Sterilized Hoagland solution
**OFSS**	**OF soil**	**Bacteria and fungi**	**50** **g soil was mixed with 250** **ml Hoagland solution, shaker for 1** **h, and centrifuged at 569** **g for 5** **min**
**BFSS**	**BF soil**		
OFSS-FF	OF soil	Bacteria	OFSS or BFSS was further centrifuged at 20510 g for 15 min, and filtered through 3 μm pore size membrane
BFSS-FF	BF soil		
**OFSS-MF**	**OF soil**	**None**	**OFSS or BFSS was further centrifuged at 20510** **g for 15** **min, and filtered through 0.45 μm pore size membrane**
**BFSS-MF**	**BF soil**		

BFSS and OFSS represent bacterial and fungal suspensions from the *Trichoderma*-amended bio-organic fertilizer and organic fertilizer treatments, respectively. BFSS-FF and OFSS-FF indicate bacterial suspensions from these same treatments, while BFSS-MF and OFSS-MF represent microbe-free suspensions from these same treatments.

### Fungal strain isolation and identification

Seventy fungal strains each were isolated from the OF and BF treatments using PDA medium (supplemented with 400 μg mL^−1^ chloramphenicol) from the third-season field. The isolates were then identified based on the morphological characteristics and ITS sequencing. For the latter, total genomic DNA of each isolate was extracted using the QIAamp DNA FFPE Tissue Kit (Qiagen, Germany), according to the manufacturer’s instructions. Genomic DNA served as template for amplification of the internal transcribed spacer (ITS) regions using the primers of ITS1: TCCGTAGGTGAACCTGCGG and ITS4: TCCTCCGCTTATTGATATGC^34^. PCR thermocycling was performed using the following conditions: initial denaturing at 94 °C for 3 min, and 30 cycles of denaturing at 94 °C for 30 s, annealing at 56 °C for 1 min, and extension at 72 °C for 1 min, with a final extension at 72 °C for 10 min. The amplified fragment of each isolate was sent for Sanger sequencing. The obtained sequences (dataset S1) were aligned with MUSCLE that integrated in the MEGA5 software and were grouped to phylotypes for reducing redundancy^35^. Unique phylotypes were subjected to the sequence similarity search tool of BLASTn against the NCBI GenBank database (June 2020). A most similar species was assigned to the query strain when sequences were found to be identical to the known reference strains, if applicable.

### Effects of selected fungal strains on plant growth

An additional pot experiment was performed to evaluate the effects of selected fungal strains on plant growth. We focused specifically on two fungal strains, BF21 and BF68 because they represented the two dominant fungal species, *A. tamarii* and *A. niger*. The pot experiment contained the following seven treatments: OFS, soil collected from OF treatment and amended with no fungal strain; BF68, soil collected from OF treatment and amended with BF68; BF21, soil collected from OF treatment and amended with the BF21; 4742, soil collected from OF treatment and amended with the NJAU 4742; BF68 + 4742, soil collected from OF treatment and amended with the mixture of BF68 and NJAU 4742 (1:1); BF21 + 4742, soil collected from OF treatment and amended with the mixture of BF21 and NJAU 4742 (1:1); BFS, soil collected from BF treatment and amended with no fungal strain. Each pot (500 g DW soil) contained one cucumber seedling and received the same number of fungal spores (10^6^ CFU/g soil DW) as per treatments described above. Each treatment got four replicates. Fifteen days after transferring the seedlings, fresh weight of the cucumber plants was measured.

### DNA extraction, Illumina Miseq sequencing, and Real-Time PCR quantification

DNA extraction and Illumina Miseq sequencing were also described in detail by Guo et al.^19^. The copies of *Trichoderma guizhouense* NJAU 4742 were quantified by Real-Time PCR using primers (ITS1 S and ITS1 R) with the strain-specific TaqMan probe ITS TM-037 Fam: 5′-FAM-AACTCTTTTTGTATACCCCCTCGCGGGT-TAMRA-3′ (FAM: 6-carboxyfluorescein, TAMRA: 6-carboxy-tetramethylrhodamine) (TaKaRa) according to Cai et al.^20^.

### Bioinformatic and statistical analysis

Significant differences in treatments were determined by independent-sample T-test and one-way Anova test with Tukey in the IBM SPSS 25.0 software program (SPSS Inc., USA). Alpha diversity including Shannon index, Chao1 estimator, and Pielou index were analyzed and compared in R environment (version 3.6.6) with package “vegan”. Principal coordinate analysis (PCoA) based on the Bray–Curtis distance was applied to assess the variations of bacterial and fungal community structures and permutational analysis of variance (PERMANOVA) was performed to evaluate the significant differences in community structures. Random forest (RF) analysis was used to identify the main predictors on growth-promoting and community construction and estimate the importance of these predictors respectively. To estimate the importance of these diversity and structure indices, we used percentage increases in the MSE (mean squared error) of variables: higher MSE% values imply more important variables^36^. RF analysis was implemented in R. The significance of each predictor was assessed with the “rfPermute” package and significance of the models were evaluate by using the “A3” package. Hierarchical cluster analysis was performed in MEGA (version 7.0.21) and sequence alignment was performed in web BLAST of NCBI (https://www.ncbi.nlm.nih.gov).

## Supplementary information

Supplementary file

Dataset 1

## Data Availability

The raw sequences of field and pot experiments were deposited in the NCBI Sequence Read Archive database with the accession number PRJNA796467 and SRP082481, respectively. Sequences of seventy fungal strains screened for alignment can be found in the additional file.

## Code availability

The scripts can be found in the Supplementary material.
