# Supplementary material for: Trichoderma-amended biofertilizer stimulates soil resident Aspergillus population for joint plant growth promotion
Source: NPJ Biofilms Microbiomes. 2022 Jul 12;8:57. doi: 10.1038/s41522-022-00321-z (PMC9279317; doi:10.1038/s41522-022-00321-z)
Supplement: Supplementary file 1 — Supplementary file [file 41522_2022_321_MOESM1_ESM.pdf]

Supplementary Information

Supplementary Table 1 Fungal strains isolation from BF and OF soil samples.

Genus	Most similar species	BOF	BBF
Trichoderma	*Trichoderma harzianum*	0	3
	T. asperellum	5	2
Fusarium	*Fusarium chlamydosporum*	1	0
	F. oxysporum	3	0
	F. phaseoli	1	0
	F. solani	0	1
Aspergillus	*Aspergillus flavus*	0	3
	A. flavipes	1	0
	A. niger	31	29
	A. tamarii	4	17
	A. tubingensis	0	3
	A. terreus	1	0
	A. fumigatus	3	1
Penicillium	*Penicillium griseofulvum*	0	1
	P. madriti	1	0
	P. oxalicum	2	0
	P. viridicatum	1	0
Mucor	*Mucor circinelloides*	1	0
Rhinocladiella	*Rhinocladiella similis*	1	0
Rhizopus	*Rhizopus oryzae*	1	2
**Unidentified**	**-**	13	8

BBF and BOF represent bulk soils samples from the *Trichoderma*-amended bio-organic fertilizer and organic fertilizer treatments, respectively.

Supplementary Figure 1 Growth of *Arabidopsis* cultivated with none-sterilized soil (A) and γ-sterilized soil (B) of different treatments. Soil collected from OF and BF treatments defined as OFS and BFS. Soil collected and then γ-sterilized from OF and BF treatments defined as SOFS and SBFS. An asterisk indicates a statistically significant difference (Turkey's test, **p* < 0.05, ***p* < 0.01, ****p* < 0.001) between SOFS and SBFS treatments.

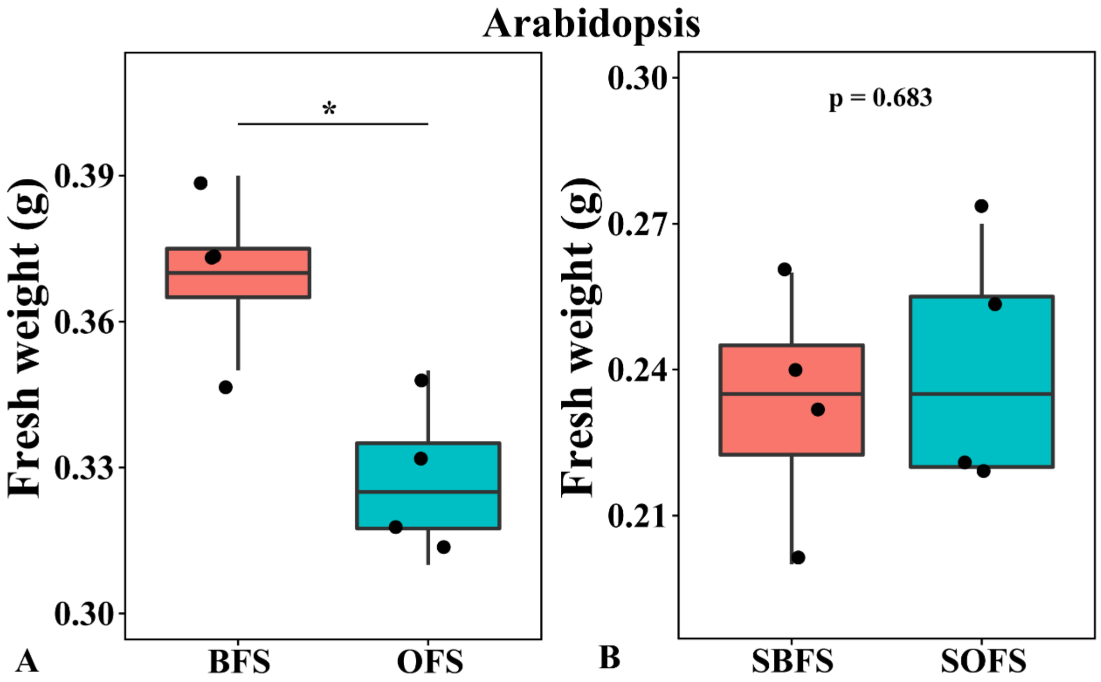

Supplementary Figure 2 Effects of different treatments on soil alpha-diversity of bacteria (A) and fungi (B) in the field experiment. The index of Shannon, Chao1 and Pielou respectively represent diversity, richness and evenness of microbial community. An asterisk indicates a statistically significant difference (*p* < 0.05, Turkey's test) between different treatments.

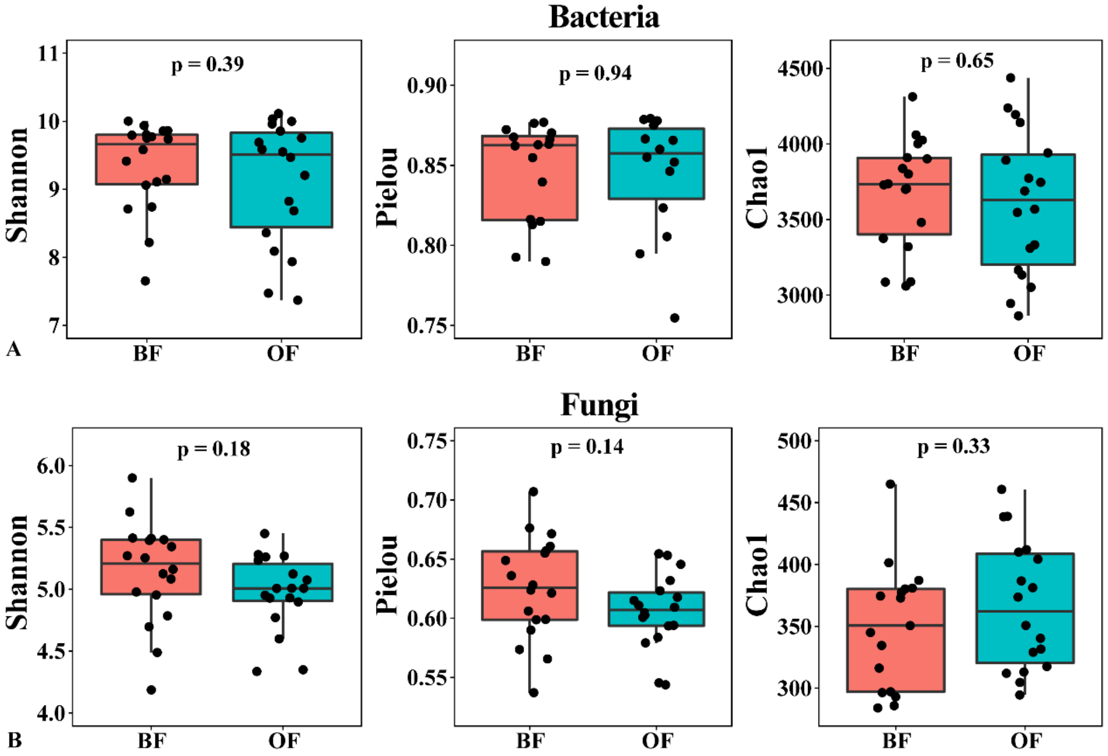

Supplementary Figure 3 Principal coordinate analysis (PCoA) of bacterial(A) and fungal (B) community composition based on bray-curtis distance of bulk soil samples in field experiment. Differences in bacterial and fungal beta diversity of different treatments soils were determined by Permutational multivariate analysis of variance (PERMANOVA). (**p* < 0.05, ***p* < 0.01 and ****p* < 0.001.)

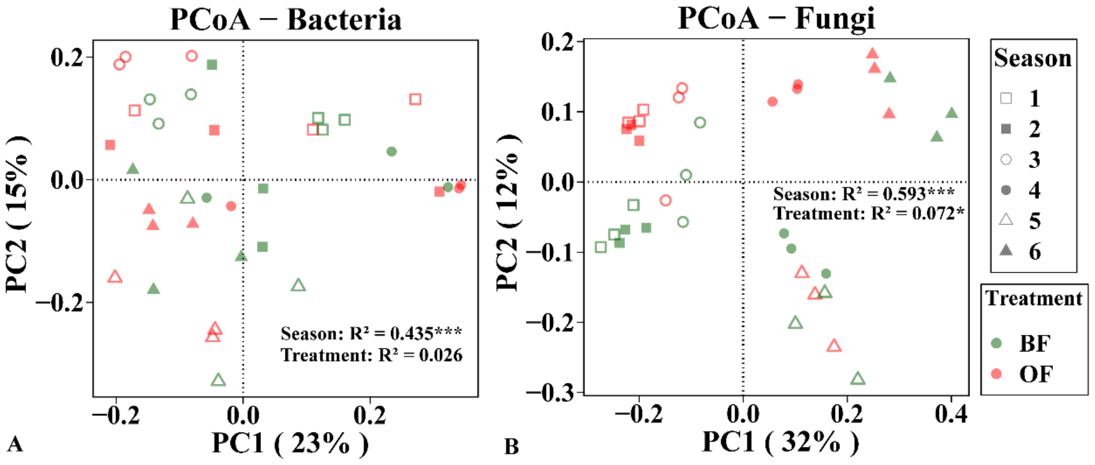

Supplementary Figure 4 Principal coordinate analysis (PCoA) of bacterial(A) and fungal (B) community composition based on bray-curtis distance of bulk and rhizosphere soil samples in pot experiment. Differences in bacterial and fungal beta diversity of different treatments soils were determined by Permutational multivariate analysis of variance (PERMANOVA). (**p* < 0.05, ***p* < 0.01 and ****p* < 0.001.)

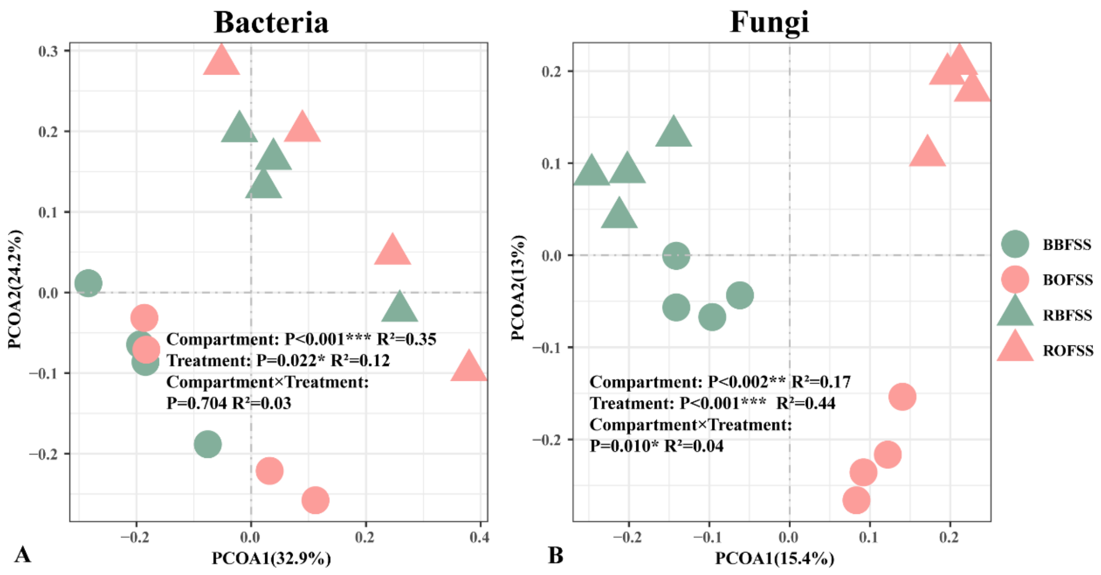

Supplementary Figure 5 Stamp analyses based on Benjamini-Hochberg FDR of the top 20 most abundant bulk (A) and rhizosphere (B) fungal genera. BFSS and OFSS represent fungal suspensions from the Trichoderma-amended bio-organic fertilizer and organic fertilizer treatments, respectively. BBFSS and BOFSS represent bulk soils samples from BFSS and OFSS, respectively, while RBFSS and ROFSS indicate rhizosphere samples from these same treatments.

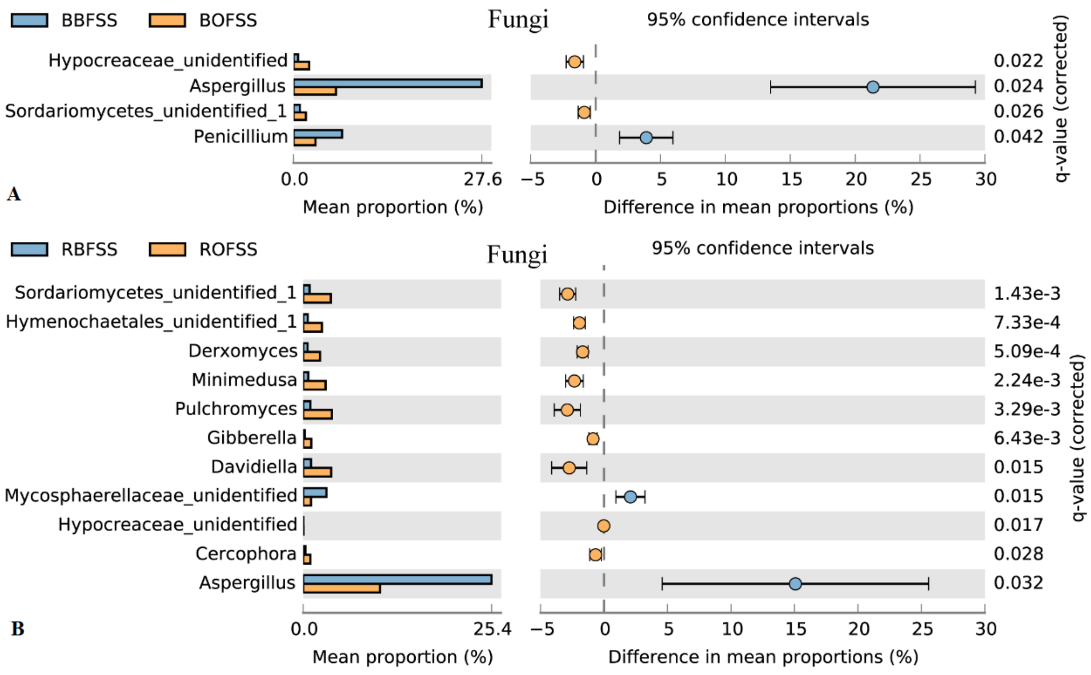

Supplementary Figure 6 The relative abundance of *Fusarium* in BF and OF treatments in third-season field experiment. Significance levels are as follows: *P < 0.05 and **P < 0.01.

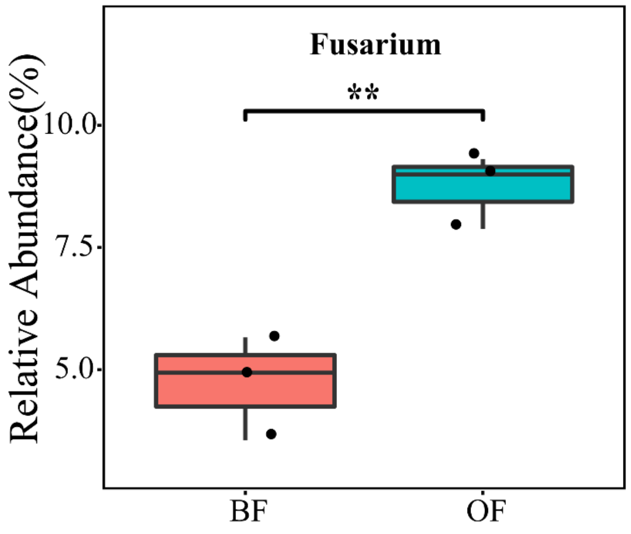

Supplementary Scripts:

###The script details of the UPARSE pipeline:

```
usearch -fastq_mergepairs *_R1.fq -fastqout merged.fq -relabel @
usearch -fastq_filter merged.fq -fastq_truncle n 200 -fastq_maxee 0.5 -fastaout filtered.fa
usearch -fastx_uniques filtered.fa -fastaout uniques.fa -sizeout -relabel Uniq
usearch -cluster_otus uniques.fa -otus otus.fa -uparseout out.up -relabel OTU -minsize 2
usearch -otutab merged.fq -otus otus.fa -otutabout otutab_raw.txt
```

###The script of PCoA

```
library(vegan)
library(ape)
library(ggplot2)
library(ggrepel)
library(plyr)
data <- read.table("otu_b_ob.txt", head=TRUE,sep="\t",row.names = 1)
groups <- read.table("otu_group_ob.txt",sep = "\t",header = F,colClasses = c("character"))
groups <- as.list(groups)
data <- t(data)
data[is.na(data)] <- 0
data <- vegdist(data,method = "bray")
pcoa<- pcoa(data, correction = "none", rn = NULL)
PC1 = pcoa$vectors[,1]
PC2 = pcoa$vectors[,2]
PC3 = pcoa$vectors[,3]
plotdata <- data.frame(rownames(pcoa$vectors),PC1,PC2,PC3,groups$V2,groups$V3)
colnames(plotdata) <-c("sample","PC1","PC2","PC3","season","treatment")
pich=c(0,15,1,16,2,17)
cbbPalette <-
c("#006000","#FF0000","#66B3FF","#FF9797","#FF5151","#EA0000","#D0D0D0","#ADA
DAD","#7B7B7B")
pc1 <-floor(pcoa$values$Relative_eig[1]*100)
pc2 <-floor(pcoa$values$Relative_eig[2]*100)
pc3 <-floor(pcoa$values$Relative_eig[3]*100)
ggplot(plotdata, aes(PC1, PC2)) +
```

```
geom_point(aes(shape=season,fill=treatment,colour=treatment),size=4,alpha=0.5)+
#geom_polygon(data=hulls,aes(fill = treatment),alpha = 0.4)+
scale_shape_manual(values=pich)+
scale_colour_manual(values=cbbPalette)+
labs(title="PCoA - Bacteria") +
xlab(paste("PC1 ( ",pc1,"%"," )",sep="")) +
ylab(paste("PC2 ( ",pc2,"%"," )",sep=""))+
geom_vline(aes(xintercept = 0),linetype="dotted")+
geom_hline(aes(yintercept = 0),linetype="dotted")+
theme(panel.background = element_rect(fill='white', colour='black'),
        panel.grid=element_blank(),
        text=element_text(family="serif", size=15),
        axis.title = element_text(color='black',size=28),
        axis.ticks.length = unit(0.4,"lines"), axis.ticks = element_line(color='black'),
        #axis.line = element_line(colour = "black"),
        axis.title.x=element_text(colour='black', size=28),
        axis.title.y=element_text(colour='black', size=28),
        axis.text=element_text(colour='black',size=25),
        legend.title=element_text(colour = "black",size = 25,face = "bold"),
        legend.text=element_text(size=23),
        legend.key=element_blank(),
        legend.background = element_rect(colour = "black"),
        legend.key.height=unit(1,"cm"))+
theme(plot.title = element_text(size=30,colour = "black",hjust = 0.5,face = "bold"))
```

###The script of generalize linear model

```
library(nutshell)
library(nutshell.bbdb)
library(MASS)
aggregate1 <- read.csv("yield_ob.csv")
head(aggregate1)
```

```
lm0<-lm(yield~1, data=aggregate1)

lm.ste<-step(lm0, scope = ~ba+fa+bb+fb, k=5)

add1(lm0, scope = ~ba+fa+bb+fb, test="F")

fit <-lm(yield~ba+fa+bb+fb,data=aggregate1)

anova(fit)

summary(fit)

AIC(fit)

relweights <- function(fit, ...) {

  R <- cor(fit$model)

  nvar <- ncol(R)

  rxx <- R[2:nvar, 2:nvar]

  rxy <- R[2:nvar, 1]

  svd <- eigen(rxx)

  evec <- svd$vectors

  ev <- svd$values

  delta <- diag(sqrt(ev))


  # correlations between original predictors and new orthogonal variables

  lambda <- evec %*% delta %*% t(evec)

  lambdasq <- lambda^2

  beta <- solve(lambda) %*% rxy

  rsquare <- colSums(beta^2)

  rawwgt <- lambdasq %*% beta^2

  import <- (rawwgt/rsquare) * 100

  lbls <- names(fit$model[2:nvar])

  rownames(import) <- lbls

  colnames(import) <- "Weights"

  barplot(t(import), names.arg = lbls, ylab = "% of R-Square",

          xlab = "Predictor Variables", main = "Relative Importance of Predictor
Variables",

          sub = paste("R-Square = ", round(rsquare, digits = 3)),
```

```
                ...)
    return(import)
}
relweights(fit, col = "lightgrey")
library(ggplot2)
df= read.csv("importance.csv",header = TRUE)
head(df)
glmPalette <- c("#0072E3","#FF2D2D","#0072E3","#FF2D2D")
p=ggplot(data = df, aes(x = treatment, y = Weights, fill = treatment)) +guides(fill=F)+
    geom_bar(stat = "identity", position = "dodge",width = 0.5) +
    theme_bw()+theme(
        panel.background=element_blank(),
        text=element_text(family="serif", size=18),
        panel.grid=element_blank(),
        axis.title.x = element_text(size=18,colour = "black",face = "bold"),
        axis.title.y = element_text(size=18, angle=90,colour = "black",face = "bold"),
        axis.text.x=element_text(size=15,colour = "black",face = "bold"),
        axis.text.y=element_text(size=15,colour = "black",face = "bold"),
        legend.direction = "horizontal",
        legend.title = element_blank(),
        legend.text = element_text(size=15,colour = "black",face = "bold"),
        legend.key.size = unit(1.2, "line"),
        legend.key = element_blank() )
p
```

samples	yield	ba	fa	bb	fb
BF1-1	44.575	-0.03055	0.107352	0.081852	-0.09278
BF1-2	46.0875	-0.06698	-0.02172	0.097866	-0.03281
BF1-3	45.33125	-0.01408	0.056463	0.100933	-0.07501
BF2-1	54	0.027932	0.02807	-0.10931	-0.08671
BF2-2	54.25	0.01885	-0.00057	-0.01426	-0.06537
BF2-3	54.125	0.046255	-0.01525	0.187626	-0.06793
BF3-1	49.325	0.029852	-0.20828	0.139303	-0.0569
BF3-2	46.3125	0.04984	0.046626	0.131131	0.010024
BF3-3	47.875	0.081679	-0.0593	0.091511	0.08463
BF4-1	53.0125	-0.06583	0.106338	-0.01193	-0.09487
BF4-2	52.425	0.033232	0.077725	-0.0292	-0.07329
BF4-3	50.25	-0.07561	0.093784	0.046242	-0.13065
BF5-1	47.25	-0.05137	-0.05067	-0.32852	-0.20237
BF5-2	50.875	0.010858	-0.01101	-0.17405	-0.2819
BF5-3	45.5	0.036895	-0.03306	-0.03111	-0.15838
BF6-1	49.675	0.008024	-0.13387	-0.17947	0.147401
BF6-2	50.3125	0.004312	-0.01118	-0.12624	0.09662
BF6-3	49.6	0.04401	0.079554	0.015954	0.063146
OF1-1	36.5	-0.08216	0.090066	0.131487	0.084677
OF1-2	41.375	-0.07268	0.049969	0.113016	0.086706
OF1-3	38.9375	-0.00747	0.066268	0.081913	0.103025
OF2-1	48.375	-0.0738	0.074886	0.056828	0.058814
OF2-2	44.375	0.051449	0.033304	0.080751	0.075363
OF2-3	46.375	-0.03997	-0.05594	-0.0193	0.08139
OF3-1	41.3125	0.014218	-0.07808	0.188085	0.1204
OF3-2	40.1875	0.029967	-0.0602	0.200417	0.133564
OF3-3	43.4625	0.003429	-0.02563	0.201957	-0.02635
OF4-1	43.6	0.103513	-0.02424	-0.04311	0.114601
OF4-2	42.875	-0.04178	0.036714	-0.00833	0.13916

OF4-3	44.275	-0.08435	0.0623	-0.01404	0.132634
OF5-1	42.1875	-0.11048	-0.10226	-0.16004	-0.16066
OF5-2	43.625	0.017869	-0.12537	-0.24471	-0.13022
OF5-3	41.1875	-0.00676	-0.0608	-0.25679	-0.23515
OF6-1	42.8375	0.067111	-0.0077	-0.07192	0.161146
OF6-2	42.1875	0.071039	0.011551	-0.04915	0.181668
OF6-3	38.8625	0.073556	0.064151	-0.07542	0.096385

###The script of random forest

```
library(rfPermute)

library(ggplot2)

library(A3)

otu <- read.delim('yield-obotu.txt', row.names = 1)

set.seed(1000)

otu_rfP <- rfPermute(Yield~., data = otu, importance = TRUE, ntree = 500, nrep = 1000, num.cores = 8)

otu_rfP

importance_otu.scale <- data.frame(importance(otu_rfP, scale = TRUE), check.names = FALSE)

importance_otu.scale

importance_otu.scale.pval <- (otu_rfP$pval)[ , , 1]

importance_otu.scale.pval

plot(rp.importance(otu_rfP, scale = TRUE))

importance_otu.scale <- importance_otu.scale[order(importance_otu.scale$'%IncMSE', decreasing = TRUE), ]

importance_otu.scale$OTU_name <- rownames(importance_otu.scale)

importance_otu.scale$OTU_name <- factor(importance_otu.scale$OTU_name, levels = importance_otu.scale$OTU_name)

p1 <- ggplot() +

    geom_col(data = importance_otu.scale, aes(x = OTU_name, y = `%IncMSE`), width = 0.7, fill = '#00AEAE', color = NA) +

    labs(title = NULL, x = NULL, y = 'Increase in MSE (%)', fill = NULL) +
```

```
    theme(panel.grid = element_blank(), panel.background = element_blank(), axis.line = element_line(colour = 'black')) +

    theme(axis.text.x = element_text(angle = 45, hjust = 1,color='black'),

          axis.text.y=element_text(color="black",size=24),

          text=element_text(family="serif", size=24)) +

    scale_y_continuous(expand = c(0, 0), limit = c(0, 11))

  p1

  for (OTU in rownames(importance_otu.scale)) {

    importance_otu.scale[OTU,'%IncMSE.pval'] <- importance_otu.scale.pval[OTU,'%IncMSE']

    if (importance_otu.scale[OTU,'%IncMSE.pval'] >= 0.05) importance_otu.scale[OTU,'%IncMSE.sig'] <- ''

    else if (importance_otu.scale[OTU,'%IncMSE.pval'] >= 0.01 & importance_otu.scale[OTU,'%IncMSE.pval'] < 0.05) importance_otu.scale[OTU,'%IncMSE.sig'] <- '*'

    else if (importance_otu.scale[OTU,'%IncMSE.pval'] >= 0.001 & importance_otu.scale[OTU,'%IncMSE.pval'] < 0.01) importance_otu.scale[OTU,'%IncMSE.sig'] <- '**'

    else if (importance_otu.scale[OTU,'%IncMSE.pval'] < 0.001) importance_otu.scale[OTU,'%IncMSE.sig'] <- '***'

  }

  p1 <- p1 +

    geom_text(data = importance_otu.scale, aes(x = OTU_name, y = `%IncMSE`, label = `%IncMSE.sig`), nudge_y = 0.5,family="serif",size=8)

  p1

  p1 <- p1 +

    annotate('text', label = 'Yield', x = 10, y = 10, size = 10,family="serif") +

    annotate('text', label = 'R2=0.41,p<0.001', x = 10, y = 9, size = 6,family="serif")

    #annotate('text', label = sprintf('italic(R^2) == %.2f', 0.576), x = 10.5, y = 9.5, size = 6, parse = TRUE,family="serif")

  p1



  set.seed(1000)

  otu_forest.pval <- a3(Yield~., data = otu, model.fn = randomForest, p.acc = 0.001, model.args = list(importance = TRUE, ntree = 500))

  otu_forest.pval
```

```
p1 <- p1 +
    annotate('text', label = sprintf('italic(P) < %.3f', 0.001), x = 10.5, y = 8.5, size = 6, parse = TRUE,family="serif")
p1
```
